# One in a Million: A Woman Presenting with Unilateral Painful Ophthalmoplegia

**DOI:** 10.5811/cpcem.2582

**Published:** 2024-04-17

**Authors:** Kevin Bennett, Eric Boccio

**Affiliations:** Memorial Healthcare System, Department of Emergency Medicine, Hollywood, Florida

**Keywords:** *Tolosa-Hunt syndrome*, *ophthalmoplegia*, *cavernous sinus*

## Abstract

**Case Presentation:**

A 52-year-old female presented to the emergency department with four days of right periorbital pain, ipsilateral temporal headache, diplopia, and photophobia. Physical examination of the right eye revealed painful ophthalmoplegia, cranial nerves III and VI paresis, increased intraocular pressure, and mild proptosis. Magnetic resonance venogram and magnetic resonance imaging orbits with contrast demonstrated an abnormal signal surrounding the right cavernous sinus/petrous apex. Tolosa-Hunt syndrome (THS) was diagnosed. Per neurology recommendations, the patient was placed on a steroid regimen over the course of three weeks. She was discharged on hospital day nine following resolution of symptoms. She had no recurrence of symptoms or residual deficits noted at her two-week follow-up appointment.

**Discussion:**

With an estimated annual incidence of one case per million, THS is a sinister etiology of unilateral headache, painful ophthalmoplegia, and oculomotor palsy. Tolosa-Hunt syndrome is caused by granulomatous inflammation in the cavernous sinus and is highly responsive to corticosteroids. Magnetic resonance imaging studies of the cavernous sinus and orbital apex are highly sensitive for THS and characteristically show enlargement and focal-enhancing masses within the affected cavernous sinus.

CPC-EM Capsule What do we already know about this clinical entity?

*Tolosa-Hunt syndrome (THS) is a rare cause of headache, painful ophthalmoplegia, and oculomotor palsy with characteristic findings on MRI.*

What is the major impact of the image?

*Magnetic resonance imaging of the orbits demonstrating focal enhancement of the affected cavernous sinus and petrous apex is characteristic of THS.*

How might this improve emergency medicine practice?

*Recognizing the appropriate radiographic modality and findings of THS will lead to earlier diagnosis and therapeutic management while avoiding unnecessary tests.*

## CASE PRESENTATION

A 52-year-old Black female with history of iron deficiency anemia presented to the emergency department with four days of right periorbital pain, ipsilateral temporal headache, diplopia, and photophobia. Incidentally, the patient reported sinus congestion one month prior that had responded to fluticasone propionate intranasal. Triage vital signs were within normal limits. Physical examination of the right eye demonstrated blurry vision with positive light perception, pupil equal, round, and reactive to light, an increased ocular pressure of 39 millimeters of mercury (mm Hg) (reference range: 10–21 mm Hg), and mild proptosis. Cranial nerves III and VI paresis was observed resulting in ptosis and impaired abduction, adduction, and upward and downward gaze. The patient did not tolerate a fundoscopic examination and voluntarily kept her right eyelid closed due to photophobia.

**Image 1.**
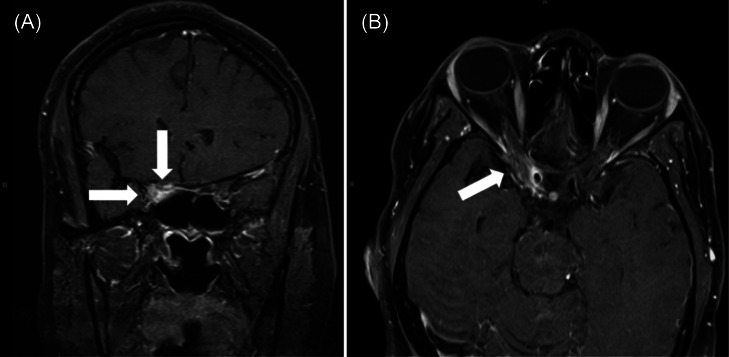
Coronal (A) and axial (B) slices of magnetic resonance imaging orbits with contrast demonstrating an abnormal signal surrounding the right cavernous sinus and petrous apex suspicious for Tolosa-Hunt syndrome (arrows).

Laboratory tests, including a complete blood count, basic metabolic panel, and urine pregnancy, were unremarkable. The electrocardiogram demonstrated a normal sinus rhythm. Computed tomography (CT) brain and CT angiogram brain and carotids revealed a cavernous sinus filling defect concerning for sinus venous thrombosis, and a heparin infusion was initiated. Magnetic resonance venogram and magnetic resonance imaging orbits with contrast demonstrated an abnormal signal surrounding the right cavernous sinus and petrous apex (Image 1).

Tolosa-Hunt syndrome (THS) was diagnosed, and the heparin infusion was discontinued. Neurology was consulted; 250 milligrams (mg) solumedrol intravenous (IV) was administered, and the patient was admitted to the intermediate medical care unit. She underwent a lumbar puncture, which demonstrated a normal opening pressure and negative cerebrospinal fluid analysis. Per neurology recommendations, she was placed on a steroid regimen consisting of three days of 250 mg solumedrol IV daily followed by one week of 80 mg prednisone per os daily followed by a taper of 20 mg per week over the course of three weeks. The patient was discharged on hospital day 9 following resolution of symptoms. She had no recurrence of symptoms nor residual deficits noted at her two-week follow-up appointment.

## DISCUSSION

With an estimated annual incidence of one case per million, THS is a sinister etiology of unilateral headache, painful ophthalmoplegia, and oculomotor palsy.^1^^,^^2^ The reported average age of onset is 41 years old. (Pediatric cases have only rarely been described).^3^ Granulomatous inflammation involving lymphocytes and plasma cells increases the pressure within the cavernous sinus and may result in compression of cranial nerves III, IV, and VI as well as the sympathetic plexus surrounding the internal carotid artery.^4^^,^^5^ While the etiology is presumed to be idiopathic, there is a strong association with autoimmune disorders such as systemic lupus erythematosus and sarcoidosis.^6^^,^^7^ Magnetic resonance imaging studies of the cavernous sinus and petrous apex are highly sensitive for THS and characteristically show enlargement and focal-enhancing masses within the affected cavernous sinus.^8^^,^^9^ While there is no consensus nor are there guidelines for managing symptoms attributed to THS, high-dose steroids are considered first line.^10^ Rapid response to steroid therapy is the hallmark of THS, and patients typically recover with no residual deficits.^11^
